# Strategies Developed by *Toxoplasma gondii* to Survive in the Host

**DOI:** 10.3389/fmicb.2019.00899

**Published:** 2019-04-26

**Authors:** Wanbo Zhu, Jingyang Li, Faustina Pappoe, Jilong Shen, Li Yu

**Affiliations:** ^1^ Department of Microbiology and Parasitology, Anhui Provincial Laboratory of Microbiology and Parasitology, Anhui Key Laboratory of Zoonoses, Anhui Medical University, Hefei, China; ^2^ Graduate School of Affiliated Anhui Provincial Hospital, Anhui Medical University, Hefei, China; ^3^ The Clinical Laboratory of the Third People’s Hospital of Heifei, Hefei, China; ^4^ Department of Microbiology and Immunology, School of Medical Sciences, University of Cape Coast, Cape Coast, Ghana

**Keywords:** *Toxoplasma*, intracellular parasites, cell invasion, immune evasion, autophagy

## Abstract

One of the most successful intracellular parasites, *Toxoplasma gondii* has developed several strategies to avoid destruction by the host. These include approaches such as rapid and efficient cell invasion to avoid phagocytic engulfment, negative regulation of the canonical CD40-CD40L-mediated autophagy pathway, impairment of the noncanonical IFN-γ-dependent autophagy pathway, and modulation of host cell survival and death to obtain lifelong parasite survival. Different virulent strains have even evolved different ways to cope with and evade destruction by the host. This review aims to illustrate every aspect of the game between the host and *Toxoplasma* during the process of infection. A better understanding of all aspects of the battle between *Toxoplasma* and its hosts will be useful for the development of better strategies and drugs to control the parasite.

## *Toxoplasma* and Host Immunity

*Toxoplasma gondii* is an obligate intracellular protozoan parasite with a unique apical complex composed of specialized cytoskeletal and secretory organelles, including micronemes, rhoptries, and dense granules. *T. gondii* is the single species in the genus *Toxoplasma*, although recent studies revealed that it has many genetic types distributed across the continents. In North America and Europe, populations of *T. gondii* are dominated by three archetypal types (I, II, or III) (Darde et al., 1992; Howe and Sibley, 1995; Howe et al., 1997; Ajzenberg et al., 2002), which vary substantially in virulence. Type I strains are highly virulent to mice with an LD100 as low as a single parasite, whereas strains of types II and III are less virulent (LD100 > 1,000) (Sibley and Boothroyd, 1992; Sibley et al., 2009). Unlike in North America and Europe, strains of *T. gondii* in South America are much more genetically diverse. Chinese 1, however, has been reported to be the most common type in East Asia, especially in China (Zhou et al., 2010, 2011; Chen et al., 2011; Wang et al., 2013; Li et al., 2014).

*T. gondii* is one of the most successful parasites, capable of invading and replicating within almost all nucleated cells of warm-blooded animals including humans (Dubey, 2010). The distinctive characteristic of this parasite is its ability to induce long-term chronic infections through its interactions with the host, leading to conversion of the prolific tachyzoite stage to the quiescent bradyzoite parasite stage (Aliberti, 2005). Bradyzoite forms of the parasite are not usually harmful in immunocompetent individuals, although in immunodeficient individuals, such as tumor and AIDS patients, they reconvert into cytolytic tachyzoites, resulting in severe toxoplasmosis and distant dissemination (Ayoade et al., 2017). Despite the parasite being the subject of over 100 years of studies and efficient therapies against acute toxoplasmosis have been developed, no effective approach for chronic infection of bradyzoites has been found because of its survival strategies in the host.

Among different hosts of *T. gondii*, there are natural differences in susceptibility to the parasite, and host innate immunity is known to play a critical role in susceptibility to the infection. Most laboratory mouse strains are susceptible to infection, and therefore they have been widely used for studying immune responses against *T. gondii.* In mice, parasite profilin interacts with Toll-like receptor (TLR)11 and TLR12 on dendritic cells (DCs) to generate a potent interleukin-12 (IL-12) response in a myeloid differentiation factor 88 (MyD88)-dependent manner (Yarovinsky et al., 2005; Koblansky et al., 2013). Distinct from these stimuli, binding of the *T. gondii* protein cyclophilin-18 (C-18) to the chemokine receptor CCR5 can also activate murine DCs to produce IL-12 (Aliberti et al., 2003). As a pro-inflammatory cytokine, IL-12 stimulates NK cells, CD4^+^ T cells, and CD8^+^T cells to express interferon-γ (IFN-γ), which plays a crucial role in parasite survival during infection. IFN-γ is also produced by neutrophils in response to IL-1β and TNF. IFN-γ propagates a signal to activate the **signal transducer and activator of transcription 1 (STAT1) (see Glossary)** through the surface receptor, IFN-γR. STAT1 is critical for the host immune response against *T. gondii* infection. STAT1 upregulates the production of effector molecules such as nitric oxide (NO) and reactive oxygen species (ROS), both of which are responsible for controlling parasite invasion in mice. IFN-γ also triggers the induction of **immunity-related GTPase (IRG)** proteins and guanylate-binding proteins **(GBPs)** to damage the **parasitophorous vacuole membrane (PVM)** in mice. Additionally, an IFN-γ-independent mechanism in mouse naive macrophages was found recently, in which NADPH oxidase (Nox)-generated ROS and GBP5 restrict the replication of avirulent type III parasites (Matta et al., 2018).

Unlike mice, humans are quite resistant to *T. gondii* infection. Owing to lack of functional genes encoding the key innate sensors TLR11 and TLR12 (Roach et al., 2005), human cells have different innate immune sensing mechanisms for the parasite. A recent research revealed that the human recognition system for this parasite is based on detection of the damage-associated molecule S100A11 released from infected cells and RAGE-dependent induction of CCL2 (Safronova et al., 2019). Human cells also rely on IFN-γ and STAT1 signaling to control the replication of *T. gondii in vitro* (Ceravolo et al., 1999), although different mechanisms are explored to control intracellular parasites. Firstly, indoleamine oxidase (IDO), but not inducible nitric oxide synthase (iNOS), has been found to be an important effector to control the parasite replication in several human cell lines after IFN-γ stimulation (Pfefferkorn et al., 1986; Nagineni et al., 1996); secondly, human cells are absent in IFN-inducible full-length IRG genes and proteins (Bekpen et al., 2005; Howard et al., 2011). Although human cells express a wide repertoire of GBPs, the involvement of GBPs in the human anti-*T. gondii* response in different cell lines is controversial (Ohshima et al., 2014; Johnston et al., 2016). Therefore, it appears that two of the main mechanisms of innate resistance mediated by IRGs and GBPs in IFN-γ stimulated mouse cells are not highly active in human cells (Hakimi et al., 2017).

Rats, like humans, are quite resistant to *Toxoplasma* infection but vary in their susceptibilities depending on the rat strain (Dubey et al., 2016). The Lewis (LEW) strain exhibits a complete resistance to *Toxoplasma* infection, and its resistance is partially abrogated by neutralization of endogenous IFN-γ, which showed that IFN-γ plays some role in the resistance of rats (Sergent et al., 2005). The *Toxo1* locus containing ***Nlrp1*** (nucleotide-binding oligomerization domain, leucine-rich repeat protein 1) was identified to mediate resistance to *T. gondii* infection (Cavailles et al., 2006). *T. gondii* can activate the NLRP1 inflammasome in macrophages, leading to caspase-1-induced pyroptosis and release of the pro-inflammatory cytokines IL-1β and IL-18 (Cavailles et al., 2014; Cirelli et al., 2014). GRA35, GRA42, and GRA43 have been recently found to be required for induction of macrophage pyroptosis in Lewis rats (Wang et al., 2019).

## Efficient Invasion is Required for Parasite Survival

Because *T. gondii* is an obligate intracellular parasite, a successful and efficient cell invasion is crucial for its survival. To establish a successful invasion, *T. gondii* needs a coordinated sequential secretion of microneme and rhoptry neck proteins (RON) first to mediate the invasion. A secretion set of *T. gondii*, including ROPs and GRAs, is also involved in host modulation and long-term establishment of the parasite into the host cell. The unique invasion mechanism of *T. gondii* consists of a secretion-regulated **moving junction (MJ)**, which facilitates firm attachment between the parasite and the host plasma membranes (Alexander et al., 2005; Lebrun et al., 2005; Lamarque et al., 2011), and the glideosome, which is a specific motor system of the parasite. Formation of the MJ relies on apical membrane antigen 1 (AMA1), secreted from micronemes and translocated to the parasite’s plasma membrane, and a rhoptry neck complex (composed of RON2, RON4, RON5, and RON8 proteins), secreted from rhoptry necks and exported into the host cell (Besteiro et al., 2009; Lamarque et al., 2011; Tyler and Boothroyd, 2011; Guerin et al., 2017). RON2 exposes a short segment at the surface of the host cell that serves as a ligand for AMA1, thus forming an intimate contact to generate an irreversible interaction between the host cell and the parasite (Lamarque et al., 2011; Tonkin et al., 2011; Tyler and Boothroyd, 2011). In addition to AMA1 and RON2, *T. g*ondii has three additional AMA paralogs and two additional RON2 paralogs, which form three complexes: AMA2-RON2, AMA3-RON2L2, and AMA4-RON2L1 (Poukchanski et al., 2013; Lamarque et al., 2014; Parker et al., 2016). The exceptional molecular diversity at the parasite–host cell interface may explain why the parasite has such a wide host range. Microneme proteins like MIC4-1-6, MIC2-M2AP, and MIC3-8 complex also play a key role in adhesion that supports gliding motility and host cell invasion (Rabenau et al., 2001; Soldati et al., 2001; Meissner et al., 2002a; Wang and Yin, 2015; Gras et al., 2017). The glideosome is an actin-myosin motor complex in *T. gondii*, and it is composed of a short single-headed myosin heavy chain A (MyoA), a myosin light chain (TgMLC1), and three gliding-associated proteins, TgGAP45, TgGAP50, and TgGAP40 (Herm-Gotz et al., 2002; Meissner et al., 2002b; Frenal et al., 2010). The connection between the glideosome and the MJ complex was previously thought to be mediated by aldolase (Jewett and Sibley, 2003; Boucher and Bosch, 2015); however, it was recently identified to be mediated by glideosome-associated connector (GAC), which forms a bridge between cell surface adhesins and the actin cytoskeleton in the parasite (Jacot et al., 2016). Finally, the power produced by the motor complex enables the parasite to move to potential host cells and to enter them by pushing forward the host membrane.

After active invasion of the host cell, *Toxoplasma* dissociates from the host membrane and resides within a non-fusogenic parasitophorous vacuole (PV), which provides a physical niche for the parasite (Tahara et al., 2016). PV formation is a result of invagination of the host cell membrane and involves host cytoskeleton rearrangement, ROPs, and GRAs (Clough and Frickel, 2017). For parasite growth and proliferation, PVM is selectively permeable to small molecules and nutrients from the host through GRA17 and GRA23 (Gold et al., 2015). More importantly, as a physical barrier, the PV protects parasites from fusion with host lysosomes and endosomes, thereby enabling survival of the parasite (Mordue and Sibley, 1997; Pavlou et al., 2018).

## Cell Autophagy in Host Immunity and *Toxoplasma* Evasion

Classical autophagy, a highly evolutionarily conserved catabolic process, is a multistep lysosomal process in which intracellular damaged or superfluous proteins and organelles are engulfed by a double-membraned autophagosome and degraded after fusion with a lysosome (Deter et al., 1967; Mizushima et al., 2011; Ohsumi, 2014). Autophagy is also actively involved in the capture of intracellular parasites and routing them for destruction. This selective autophagy process is called xenophagy, a key defense mechanism against a broad range of infections (Deretic et al., 2013; Besteiro, 2018). In addition to classic autophagy, some noncanonical forms of autophagy are also involved in host defense against intracellular pathogens: LC3-associated phagocytosis (LAP) and IFNγ- inducible GTPase-mediated host defense. These processes rely on some but not all components of the autophagy machinery (Zhao et al., 2008; Lai and Devenish, 2012; Haldar et al., 2014).

**Figure 1:**
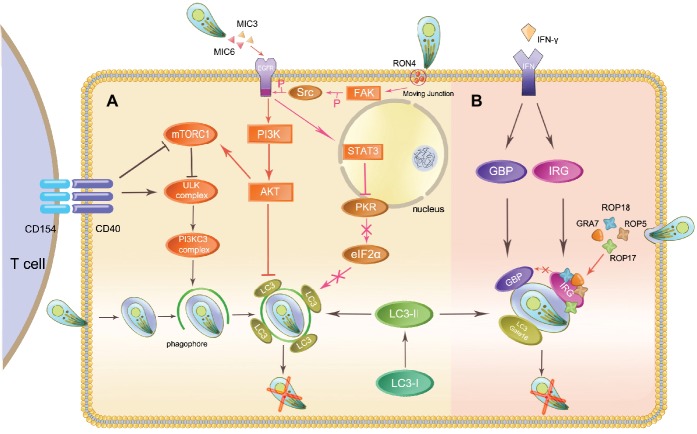
Autophagy initiates host immunity and *Toxoplasma* immune evasion. The host eliminates intracellular *Toxoplasma* through autophagy proteins in two ways: the canonical CD40-CD40L-mediated and the noncanonical IFN-γ-dependent autophagy pathways. *Toxoplasma* can evade elimination through host autophagy by following means. **(A)** In the canonical autophagy pathway, MIC3, and MIC6 secreted from *Toxoplasma* phosphorylate EGFR and activate the PI3K/AKT signaling pathway, which has an impact on LC3 function. The activation of FAK-Src through *Toxoplasma* invasion prevents this parasite from being targeted. **(B)** ROP5, ROP18, and GRA7 form a complex with ROP17 and load onto the PVM, inactivating IRGs through phosphorylation.

In *T. gondii*-infected human/murine macrophages and nonhematopoietic cells, CD40 interacts with CD40L (CD154) expressed on the surface of T cells to trigger the killing of the parasite through an autophagy-dependent pathway (Andrade et al., 2006; Portillo et al., 2010; Van Grol et al., 2013). CD40-CD40L interactions activate upstream regulators of the autophagic response such as ULK1/2 and Beclin1-PI3KC3, two important complexes required for the initiation of phagophore (an isolation membrane, precursor of the autophagosomal compartment), and then drive the recruitment of the protein LC3 to the phagophore and promote autophagosome formation (Liu et al., 2016). In contrast, IFNγ-dependent noncanonical autophagy directly binds LC3 and other **Atgs** to the PVM, leading to recruitment of two subclasses of IFNγ-inducible GTPases for parasite exposure (Selleck et al., 2015). Atgs in both canonical and noncanonical autophagy processes mediate the targeting of parasites and expose *Toxoplasma* to host immune surveillance, leading to killing of the parasite. To avoid being targeted, *Toxoplasma* developed a survival strategy against autophagy that involves immune evasion (Figure 1A).

The *Toxoplasma* micronemal proteins MIC3, and MIC6 act as ligands for EGFR, inducing phosphorylation of this protein in host cells and activating the PI3K/Akt signaling pathway. This event prevents the expression of the autophagy protein LC3 and vacuole-lysosomal fusion. Subsequently, the parasite is prevented from being targeted by the host autophagy machinery (Muniz-Feliciano et al., 2013; Wang et al., 2016). In mammalian cells, Akt phosphorylation also results in the activation of mTORC1, which negatively regulates autophagosome formation, thus inhibiting autophagy (Kim et al., 2011). During host invasion and MJ formation, *T. gondii* activates a signaling cascade downstream of FAK-Src. It inhibits the activation of the key stimulators of autophagy PKR and eIF2α, which prevent the parasite from being targeted (Portillo et al., 2017).

## Escape from GTPases-Dependent Antimicrobial Programs

As mentioned above, the IFNγ-inducible GTPase-mediated host defense plays a crucial role in the destruction of the PVM in a murine model. In IFNγ-stimulated cells, a complex containing LC3 and a subset of Atgs attaches to the PVM and recruits IFNγ-inducible GTPases, leading to vesiculation and destruction of the PVM (Lai and Devenish, 2012). Consequently, the parasites are released into the host cell cytoplasm and subsequently become exposed to the host immune system. Recent reports indicate that Gate-16 is also required for this GTPases-dependent antimicrobial program even in the absence of LC3 (Sasai et al., 2017).

Virulent type I strains of *T. gondii* can evade this autonomous host immunity mechanism but type II and III strains cannot. Rhoptry protein kinase ROP18 and pseudokinase ROP5 are known to be polymorphic and determine the virulence of different strains and thwart the IRG system (Niedelman et al., 2012). After being secreted during invasion, ROP18 phosphorylates Thr residues in the switch region 1 (SW1) on host Irga6 and Irgb6 with the cooperation of ROP5. ROP5 regulates the activity of ROP18, and additionally binds directly to monomeric IRGs to prevent oligomerization and support ROP18 phosphorylation. ROP5 is also able to associate with another PVM-associated kinase, ROP17, in phosphorylating and inactivating IRGs (Irgb6) (Etheridge et al., 2014). GRA7 binds to and increases turnover of Irga6, which makes substrates available for ROP18, thereby preventing loading of IRGs (Figure 1B; Alaganan et al., 2014; Hermanns et al., 2016). ROP5 and ROP18 are also able to inhibit GBP1 loading onto the PVM. Pseudokinase ROP54 can move to the cytoplasmic face of the PVM and restrict immune loading of GBP2 to evade the GBP2-mediated immune response (Yang et al., 2017a). In addition, ROP18 in virulent strains can phosphorylate activating transcription factor 6β (ATF6β), an important factor for DCs antigen presenting (Yamamoto et al., 2011; Yamamoto and Takeda, 2012). Phosphorylated ATF6β may be degraded and affect the antigen-presenting ability of DCs, having an impact on the interferon-inducible GTPase-mediated host defense.

## Resistance to Oxidative Stress

**Figure 2:**
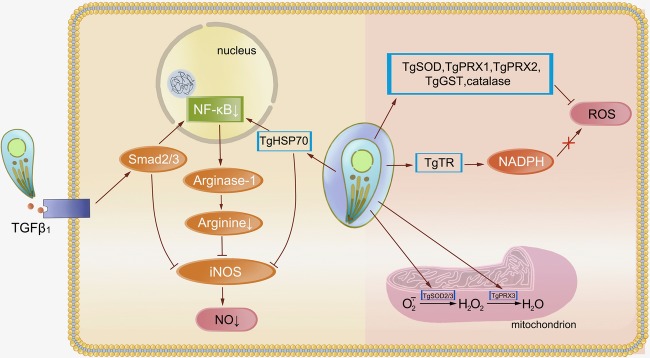
Resistance of *Toxoplasma gondii* to oxidative stress. Oxidative stress is an important way for the host to restrict *Toxoplasma* replication and prevent subsequent infection. Activation of TGFβ1 induced by *Toxoplasma* through Smad2 and Smad3 leads to destruction of iNOS. The NF-κB pathway is responsible for this decline in NO levels, whereas TgHSP70 also regulates this pathway. Cytosolic peroxiredoxins (TgPRXs) and superoxide dismutases (TgSODs) from *Toxoplasma* are able to eliminate ROS and protect the parasite against oxidative-burst injury.

Escape from GTPase-dependent antimicrobial programs is vital to *T. gondii*, and innate immune cells cytotoxicity is essential for restriction of parasite infection (Figure 2). Resistance to cytotoxicity makes sense for *T. gondii*. **Inducible nitric oxide synthase (iNOS)** expressed by mouse macrophages synthesizes NO through oxidation of L-arginine. Large amounts of NO result in parasite death but can be easily inhibited by *T. gondii* for sustained replication. The process is mediated by TGFβ1 induction through Smad2 and Smad3, leading to destruction of iNOS and actin filament (F-actin) depolymerization. After infection, high levels of arginase compete with iNOS for the same substrate, leading to a reduction of NO (Padrao Jda et al., 2014). *T. gondii* HSP70 (TgHSP70), a tachyzoite-specific protein, also contributes to downregulation of NO (Moroda et al., 2017). In human cells, the effect of iNOS is different. It acts as a pro-*Toxoplasma* host factor through the GRA15-dependent virulence mechanism in the THP-1/Huh7 coculture mode (Bando et al., 2018). Upregulation of ROS and IDO generated by many cell types leads to inhibition of parasite replication. An oxidative stress microenvironment is generated by producing hydrogen peroxide, superoxide, and hydroxyl radicals to damage the parasites or reduce tryptophan levels, preventing the parasites from rapid replication. However, *T. gondii* can subvert these mechanisms through an antioxidant network. The cytosolic peroxiredoxins TgPRX1 and TgPRX2 and mitochondrial peroxiredoxin TgPRX3 act downstream of superoxide dismutases (SODs) to detoxify hydrogen peroxide (Kwok et al., 2003). TgSOD2 and TgSOD3, similar to mitochondrial SODs, are able to eliminate the ROS produced by oxidative phosphorylation. *T. gondii* antioxidant glutathione-S-transferase (TgGST), glutaredoxin (TgGrx), and catalase also make a major contribution to decomposing superoxide anion radicals and resisting oxidative damage (Pino et al., 2007; Wang et al., 2015). *T. gondii* thioredoxin reductase (TgTR) maintains a thioredoxin-reduced state during NADPH consumption that protects parasite against oxidative-burst injury (Kim et al., 2017; Xue et al., 2017). However, this survival ability may only occur in virulent strains, whereas avirulent type III parasites are preferentially cleared by NADPH oxidase and induction of GBP5 in human cells (Matta et al., 2018).

## Regulation of Host Gene Expression by *T. Gondii*

**Figure 3:**
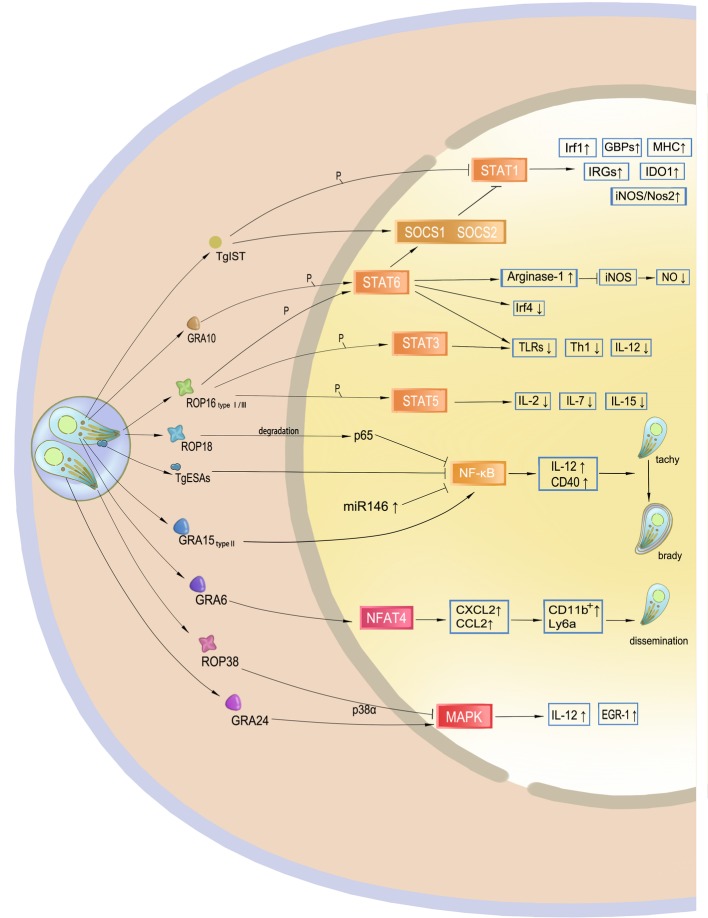
Targeting of host gene expression by *Toxoplasma gondii*. Effectors secreted by *T. gondii* manipulate host gene expression. TgIST translocates to the nucleus after *T. gondii* invasion and inhibits STAT1 by phosphorylation. GRA10 and ROP16 phosphorylate STAT3 and STAT6, mainly leading to a decrease in IL-12 and Th1 response. Additionally, phosphorylation of STAT6 results in resistance to NO. ROP18 and TgESAs block the NF-κB signaling pathway and impair CD40- and TNF-regulated parasite control of the host. However, GRA15 of type II activates this signaling pathway, which may promote cyst formation of the avirulent strain to avoid host immunity. GRA6 activates the host transcription factor NFAT4, and then attracts inflammatory cells and facilitates *T. gondii* dissemination. ROP38 and GRA24 have a diverse role in the modulation of the MAPK pathway.

Interferon-inducible cell autonomous immunity or cytotoxicity, necessary for the host to control the parasite, is based on gene expression. These signal transduction pathways enhance or regulate the overall immune response in such a way as to become subverted by *T. gondii*, constituting a major part of the *T. gondii* immune evasion mechanism (Figure 3).

STAT pathways modulate the transcription of both pro- and anti-inflammatory molecules for parasite control. The STAT1 transcription factor, the main signal transducer of IFN-γ, mediates interferon-inducible immunity but may be blocked by the *Toxoplasma* inhibitor of STAT1-dependent transcription (TgIST), resulting in blockage of interferon regulatory factor 1 (Irf1), p65 GBPs, iNOS, indoleamine 2, 3-dioxygenase 1, and major histocompatibility complex (MHC). Secreted by *T. gondii* after invasion, TgIST translocates to the nucleus and recruits the Mi-2 nucleosome remodeling and deacetylase (NuRD) complex to STAT1-dependent promoters, leading to chromatin alteration and signal blockage (Gay et al., 2016; Olias et al., 2016). In addition, this mechanism is not virulence-dependent, and thus all *T. gondii* clonal lineages equally inhibit STAT1 transcriptional activity and repress the IFN-γ response (Rosowski and Saeij, 2012). Another mechanism of IFN-γ blockage through STAT1 is dephosphorylation by suppressor of cytokine signaling phosphatase (SOCS1), an anti-inflammatory pathway that is upregulated during parasite infection.

Transcription factors STAT3 and STAT6 are associated with IL-4 and IL-6 production. After being injected into host cells, ROP16 localizes to the host nucleus through nuclear localization signals. ROP16 can disturb the host IFN-γ signal transduction and phosphorylates STAT3 and STAT6, leading to a decrease in IL-12 levels that mainly limits the protective Th1 cytokine responses and the inflammasome. Phosphorylation of STAT6 also induces the expression of arginase-1, SOCS2, and interferon regulatory factor 4 (IRF4). High levels of production of arginase-1 lead to NO degradation, resulting in resistance to host immune attacks, thereby allowing proliferation of the parasite. Although all three strain types initially induce STAT3 and STAT6 activity, only ROP16 I/III strains suppress IL-12 production in macrophages, because a single amino acid substitution in the kinase domain was identified in ROP16 II that determines the strain difference in terms of Stat3 activation (Yamamoto et al., 2009). ROP16 also induces the phosphorylation and nuclear translocation of STAT5 to generate protective immunity (Chang et al., 2015).

In addition to IRFs, CD40 and TNF, as downstream factors of NF-κB signaling pathway, are also required for parasite control. The NF-κB family of transcription factors plays a key role in host immunity against *T. gondii* because it is believed to be an evolutionarily conserved mechanism that regulates host innate and adaptive immune functions for parasite survival. ROP18 in *T. gondii* type I strains is responsible for p65 degradation and thus suppresses NF-κB activation (Du et al., 2014). NF-κB can also be inhibited by *T. gondii* excretory/secretory antigens (TgESAs), which limit the functional activity of macrophages and suppress pro-inflammatory cytokine secretion for parasite survival (Wang et al., 2017). In addition, release of GRA15 by type II strains activates the NF-κB pathway and initiates IL-12 synthesis through enhancing the expression of CD40 in infected cells (Morgado et al., 2014). Thus, type II GRA15 has the ability to effectively control acute *T. gondii* infection and promote the conversion of parasite into bradyzoites at the chronic infection stage. This mechanism is considered another *T. gondii* immune evasion strategy.

Other *T. gondii* GRA and ROP effectors contribute to host gene targeting and signaling interference. GRA6 selectively activates nuclear factor of activated T cells 4 (NFAT4), a host transcription factor, which modulates host immune responses and parasite dissemination in a strain-specific manner (Ma et al., 2014). The p38 MAP kinase pathway controls gene expression and the early immune response, such as IL-12 production, to restrain *T. gondii.* GRA24 modulates host immune responses by triggering prolonged autophosphorylation and nuclear translocation of the host cell p38 MAPK (Braun et al., 2013). Interestingly, ROP38 has an adverse influence in downregulating MAPK signaling and some other transcriptional controls without affecting parasite replication and virulence (Peixoto et al., 2010). ROP38 functions in accordance with the demands of parasite survival, including bradyzoite differentiation, or maintaining the viability of the infected host cells. GRA16 is secreted and eventually exported to the host nucleus to interact with the herpesvirus-associated ubiquitin-specific protease (HAUSP) and modulate host gene expression, such as the p53 tumor suppressor pathway, which promotes host cell survival under stress conditions. The export of the GRA16 and GRA24 effectors into host cells and their accumulation in the nucleus are mediated by the aspartyl protease TgASP5 (Curt-Varesano et al., 2016). According to a recent study, ROP17 downregulates the activation of many immune signaling pathways and transcription factors to inhibit immune responses during *T. gondii* infection in human cells (Li et al., 2019).

## Modulation of Host Cell Survival and Death for Parasite Proliferation

**Figure 4:**
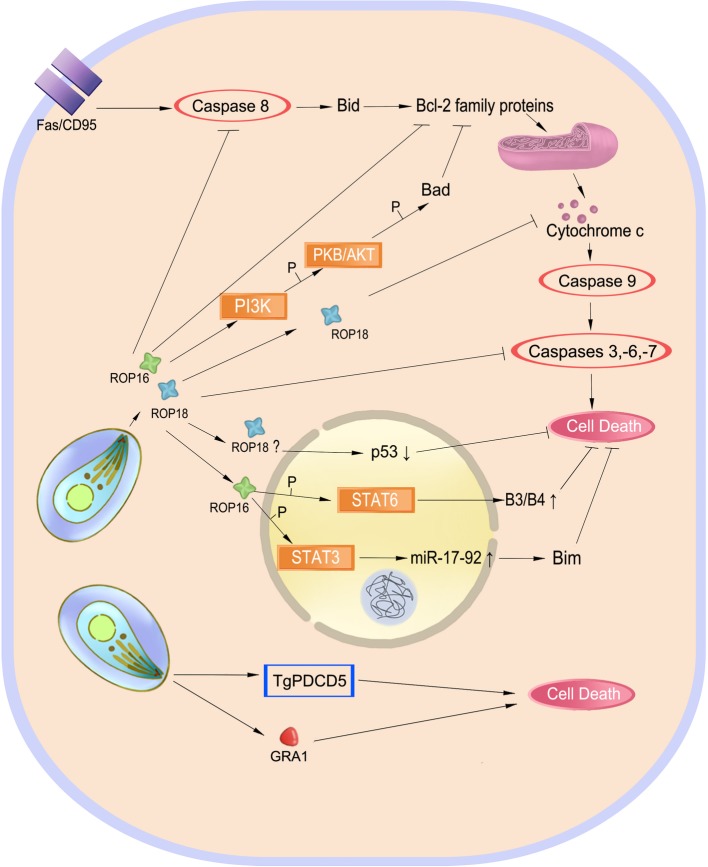
Balancing host cell survival and death. Inhibition of cell apoptosis preserves the intracellular parasite *Toxoplasma gondii*. Effectors such as ROP18 play a role in Fas/CD95-mediated apoptosis to inhibit this process. ROP16 also phosphorylates STAT3 and STAT6 to inhibit cell death. However, TgPDCD5 and GRA1 induce cell death, which may help the parasite survive by killing host’s immune cells.

Intracellular *T. gondii* depends on the sustained life of host cells for its growth, metabolism, and proliferation. Host cell death seriously threatens parasite survival, whereas apoptosis induction in some cells may suppress the immune responses against the parasite (Luder et al., 2001). Modulation of host cell survival and death is one of the strategies for *T. gondii* survival (Figure 4).

Inhibition of host cell apoptosis may preserve intracellular replication and long-term survival of the parasite. *T. gondii* infection inhibits mitochondrial apoptosis by preventing the release of cytochrome-c and phosphorylation of the pro-apoptotic Bad protein and inducing overproduction of the anti-apoptotic Bcl-2 (Hippe et al., 2008; Hwang et al., 2010; Quan et al., 2013). In infected macrophages, *T. gondii* also induces serpin B3/B4 expression through STAT6 activation and activates the STAT3-miR-17-92-Bim pathway to inhibit apoptosis (Song et al., 2012; Cai et al., 2014). In addition to blockage of cytochrome-c release, *T. gondii* targets the holo-apoptosome assembly to inhibit caspase-dependent intrinsic cell death (Graumann et al., 2015). ROP18, an important virulence determinant of the parasite, has been found to play a role in regulating apoptosis of infected cells. It inhibits host cell apoptosis by blocking the release of cytochrome-c, upregulating the ratio of Bcl-2/Bax, and inducing p53 degradation (Wu et al., 2016; Yang et al., 2017b; Xia et al., 2018).

On the other hand, induction or enhancement of apoptosis in the course of *T. gondii* infection might be beneficial for the parasite (Figure 4). Programmed cell death 5 (TgPDCD5), a protein released from *T. gondii*, enhances apoptosis of uninfected host macrophages, which appears to be a negative regulator of the immune response against the parasite (Bannai et al., 2008, 2009). Similarly, GRA1 secreted by *T. gondii* induces apoptosis of monocytes that is mediated by the TGF-β pathway (Ngo et al., 2017). Apoptosis and cell death in bystander host cells may be caused by nitric oxide and other soluble factors produced by infected cells (Nishikawa et al., 2007).

## Survival Strategies in the Chronic Infection Stage

After exponential replication as tachyzoites, the increasing host immune response and accompanying stress conditions lead *T. gondii* to differentiate into the latent bradyzoite stage that resides in tissue cysts. This stage allows the parasites to cause chronic disease owing to their ability to evade the immune system and resist common therapies. In addition, when the host immunity becomes impaired, *T. gondii* being an opportunistic pathogen may transform into rapidly replicating tachyzoites. Infectious bradyzoite cysts forming in muscle, heart, and central nervous system (CNS) tissues allow *T. gondii* to spread to a new host following predation of its former host, a part of the parasite’s life cycle.

Because of the “Trojan horse” strategy in which tachyzoites move to immune-privileged organs such as the CNS and form cysts under the regulation of multiple genes, bradyzoites within tissue cysts persist for the life of their host (Hong et al., 2017; Huang et al., 2017). Rhoptry proteins such as ROP17, ROP35, and ROP38 also promote tachyzoite conversion to encysted bradyzoite. The persistent cyst is cloaked with a glycosylated structure called the cyst wall, which provides a sturdy barrier for bradyzoite survival and infectivity for oral transmission. Cyst wall glycoprotein CST1 glycosylated by Tg.ppGalNAc-Ts confers structural rigidity in brain cysts in a mucin-like domain-dependent manner (Tomita et al., 2013, 2017). A toxoplasma nucleotide-sugar transporter (TgNST1) is also required for cyst wall glycosylation (Caffaro et al., 2013). Bradyzoite-secreted pseudokinase 1 (BPK1) is a component of the cyst wall and necessary for the growth, maintenance, and stability of tissue cysts (Buchholz et al., 2013). Several GRA proteins contribute to cyst wall formation and maintenance. A recent report demonstrated that the *T. gondii* lysosomal vacuolar compartment (TgVAC) is capable of proteolysis and maintains the viability and persistence of *T. gondii* cysts (Di Cristina et al., 2017).

## Concluding Remarks

*T. gondii* is an intracellular parasite with an efficient invasion system that avoids phagocytosis. After a successful invasion, it secretes many different effectors to disturb the canonical CD40-CD40L-mediated and the noncanonical IFN-γ-dependent autophagy, regulate host gene expression, modulate host cell survival and death, and counter host defense mechanisms. *T. gondii* needs a balance between establishing a successful infection in the host for ensuring propagation, and avoiding a too rapid multiplication that would kill the host. Additionally, as a successful intracellular parasite, obtaining nutrients from the host is also a critical survival strategy (for a more detailed discussion of nutrients, see Blume and Seeber (2018) and references therein). *T. gondii* employs different approaches in a strain-specific or host-specific manner to fight against different hosts. Striking differences in the strategies adopted by the parasite have been observed between murine and human hosts. Though anti-*Toxoplasma* responses have been extensively investigated in a mouse model, the mechanisms used by human cells to control the parasite and by the parasite to antagonize these responses remain elusive. For instance, what is the major IFN-γ-induced effector in human cells, as there are no IFNγ-inducible IRGs and a reduced GBP repertoire? What are the *Toxoplasma* virulence factors in humans, and how do they defend the PV from host destruction in human cells? As is known that ROP18 and ROP5 are the major virulence factors in strains from North America, Europe, and South America in mice, what are the virulent determinants in the atypical lineages (i.e., types 13/Chinese 1 from China, 14 from Africa, and 11 from North America), and how do these determinants fight the immune system in mice and in humans to help the parasite survive? Addressing these questions will allow a better description of the battle between *T. gondii* and its hosts, and such knowledge will be useful in the development of drugs to control the parasite.

## Author Contributions

LY and JS designed the work. JL and WZ drafted the article. LY and FP did critical revision of the article. All authors read and approved the final version of the manuscript.

### Conflict of Interest Statement

The authors declare that the research was conducted in the absence of any commercial or financial relationships that could be construed as a potential conflict of interest.

## Figures and Tables

**Table:** 

*Atgs*	Autophagy-related proteins. Eukaryotic factors participating in various stages of the autophagic process and required for autophagosome formation.
*CDPKs*	Ca^2+^-dependent protein kinases. A family of enzymes that control multiple process of parasite in motility, adhension, and egress.
*GBPs*	IFN-γ-inducible guanylate-binding proteins.
*GRAs*	Dense granule protein. Proteins secreted from the parasite dense-granule organelles and take part in most steps during parasite invasion and evasion.
*IDO*	Indoleamine oxidase. Induced by IFN-γ and degrades tryptophan, an essential nutritional amino acid for the intracellular growth of *T. gondii* in human cells.
*iNOS*	Inducible nitric oxide synthase. An IFN-γ-inducible protein that mediates parasite clearance and growth inhibition through nitric oxide production.
*IRGs*	A family of rodent IFN-γ-induced GTPases.
*LC3*	Microtubule-associated protein1 lightchain3. A mammalian autophagosomal ortholog of yeast Atg8 and contributes to major steps of autophagy. There are two isoforms of LC3, LC3-I (soluble) and LC3-II (membrane-bound).
*MICs*	Microneme proteins. Proteins secreted from the apically located parasite microneme organelles and contribute to *T.gondii* invasion and virulence.
*MJ*	Moving junction. A tight apposition between the host cell and parasite plasma membranes. Anchors the invading parasite to the host cell and facilitates parasite internalization within a parasitophorous vacuole.
*MMPs*	Matrix metalloproteases. A family of enzymes containing Ca^2+^ and Zn^2+^ in structure that participate in metabolism in extracellular matrix.
*NLRP1*	An inflammasome sensor and a member of the NLR (Nod-like receptor/nucleotide binding domain/leucine rich repeat containing) protein family.
*PV (and PVM)*	Parasitophorous vacuole. An intracellular compartment that the parasite resides in and is bounded by membrane called PVM.
*RONs*	Rhoptry neck proteins. Proteins secreted from the neck of the rhoptry organelles. A component of the moving junction that plays a central role during invasion.
*ROPs*	Rhoptry body proteins. Proteins secreted from the bulb or body of the apically located rhoptry organelles of the parasite and take part in most steps during parasite invasion and evasion.
*STAT*	Signal transducer and activator of transcription. A group of transcription factors that transmit signals from the extracellular milieu of cells to the nucleus.

## References

[ref1] Ajzenberg D. Banuls A. L. Tibayrenc M. Darde M. L. (2002). Microsatellite analysis of Toxoplasma gondii shows considerable polymorphism structured into two main clonal groups. Int. J. Parasitol. 32, 27–38. 10.1016/S0020-7519(01)00301-0, PMID: 11796120

[ref2] Alaganan A. Fentress S. J. Tang K. Wang Q. Sibley L. D. (2014). Toxoplasma GRA7 effector increases turnover of immunity-related GTPases and contributes to acute virulence in the mouse. Proc. Natl. Acad. Sci. U. S. A. 111, 1126–1131. 10.1073/pnas.1313501111 24390541 PMC3903209

[ref3] Alexander D. L. Mital J. Ward G. E. Bradley P. Boothroyd J. C. (2005). Identification of the moving junction complex of *Toxoplasma gondii*: a collaboration between distinct secretory organelles. PLoS Pathog. 1:e17. 10.1371/journal.ppat.0010017, PMID: 16244709 PMC1262624

[ref4] Aliberti J. (2005). Host persistence: exploitation of anti-inflammatory pathways by *Toxoplasma gondii*. Nat. Rev. Immunol. 5, 162–170. 10.1038/nri1547, PMID: 15662369

[ref5] Aliberti J. Valenzuela J. G. Carruthers V. B. Hieny S. Andersen J. Charest H.. (2003). Molecular mimicry of a CCR5 binding-domain in the microbial activation of dendritic cells. Nat. Immunol. 4, 485–490. 10.1038/ni915, PMID: 12665855

[ref6] Andrade R. M. Wessendarp M. Gubbels M. J. Striepen B. Subauste C. S. (2006). CD40 induces macrophage anti-*Toxoplasma gondii* activity by triggering autophagy-dependent fusion of pathogen-containing vacuoles and lysosomes. J. Clin. Invest. 116, 2366–2377. 10.1172/jci28796, PMID: 16955139 PMC1555650

[ref7] Ayoade F. Todd J. Al-Delfi F. King J. (2017). Extensive brain masses and cavitary lung lesions associated with toxoplasmosis and acquired immunodeficiency syndrome. Int. J. STD AIDS 28, 1150–1154. 10.1177/0956462417696216, PMID: 28632476

[ref8] Bando H. Lee Y. Sakaguchi N. Pradipta A. Ma J. S. Tanaka S.. (2018). Inducible nitric oxide synthase is a key host factor for *Toxoplasma* GRA15-dependent disruption of the gamma interferon-induced antiparasitic human response. MBio 9:e01738-18. 10.1128/mBio.01738-18, PMID: 30301855 PMC6178625

[ref9] Bannai H. Nishikawa Y. Ibrahim H. M. Yamada K. Kawase O. Watanabe J. (2009). Overproduction of the pro-apoptotic molecule, programmed cell death 5, in Toxoplasma gondii leads to increased apoptosis of host macrophages. J. Vet. Med. Sci. 71, 1183–1189. 10.1292/jvms.71.1183 19801898

[ref10] Bannai H. Nishikawa Y. Matsuo T. Kawase O. Watanabe J. Sugimoto C.. (2008). Programmed cell death 5 from *Toxoplasma gondii*: a secreted molecule that exerts a pro-apoptotic effect on host cells. Mol. Biochem. Parasitol. 159, 112–120. 10.1016/j.molbiopara.2008.02.012, PMID: 18406478

[ref11] Bekpen C. Hunn J. P. Rohde C. Parvanova I. Guethlein L. Dunn D. M. (2005). The interferon-inducible p47 (IRG) GTPases in vertebrates: loss of the cell autonomous resistance mechanism in the human lineage. Genome Biol. 6:R92. 10.1186/gb-2005-6-11-r92 16277747 PMC1297648

[ref12] Besteiro S. (2018). The role of host autophagy machinery in controlling *Toxoplasma* infection. Virulence 1–10. 10.1080/21505594.2018.1518102, PMID: 30269643 PMC6550551

[ref13] Besteiro S. Michelin A. Poncet J. Dubremetz J. F. Lebrun M. (2009). Export of a *Toxoplasma gondii* rhoptry neck protein complex at the host cell membrane to form the moving junction during invasion. PLoS Pathog. 5:e1000309. 10.1371/journal.ppat.1000309, PMID: 19247437 PMC2642630

[ref14] Blume M. Seeber F. (2018). Metabolic interactions between *Toxoplasma gondii* and its host. F1000Res 7, 1719–1728. 10.12688/f1000research.16021.1, PMID: 30467519 PMC6208699

[ref15] Boucher L. E. Bosch J. (2015). The apicomplexan glideosome and adhesins–structures and function. J. Struct. Biol. 190, 93–114. 10.1016/j.jsb.2015.02.008, PMID: 25764948 PMC4417069

[ref16] Braun L. Brenier-Pinchart M. P. Yogavel M. Curt-Varesano A. Curt-Bertini R. L. Hussain T.. (2013). A *Toxoplasma* dense granule protein, GRA24, modulates the early immune response to infection by promoting a direct and sustained host p38 MAPK activation. J. Exp. Med. 210, 2071–2086. 10.1084/jem.20130103, PMID: 24043761 PMC3782045

[ref17] Buchholz K. R. Bowyer P. W. Boothroyd J. C. (2013). Bradyzoite pseudokinase 1 is crucial for efficient oral infectivity of the *Toxoplasma gondii* tissue cyst. Eukaryot. Cell 12, 399–410. 10.1128/EC.00343-12, PMID: 23291621 PMC3629768

[ref18] Caffaro C. E. Koshy A. A. Liu L. Zeiner G. M. Hirschberg C. B. Boothroyd J. C. (2013). A nucleotide sugar transporter involved in glycosylation of the *Toxoplasma* tissue cyst wall is required for efficient persistence of bradyzoites. PLoS Pathog. 9:e1003331. 10.1371/journal.ppat.1003331, PMID: 23658519 PMC3642066

[ref19] Cai Y. Chen H. Mo X. Tang Y. Xu X. Zhang A.. (2014). Toxoplasma gondii inhibits apoptosis via a novel STAT3-miR-17-92-Bim pathway in macrophages. Cell. Signal. 26, 1204–1212. 10.1016/j.cellsig.2014.02.013, PMID: 24583285

[ref20] Cavailles P. Flori P. Papapietro O. Bisanz C. Lagrange D. Pilloux L.. (2014). A highly conserved Toxo1 haplotype directs resistance to toxoplasmosis and its associated caspase-1 dependent killing of parasite and host macrophage. PLoS Pathog. 10:e1004005. 10.1371/journal.ppat.1004005, PMID: 24699513 PMC3974857

[ref21] Cavailles P. Sergent V. Bisanz C. Papapietro O. Colacios C. Mas M. (2006). The rat Toxo1 locus directs toxoplasmosis outcome and controls parasite proliferation and spreading by macrophage-dependent mechanisms. Proc. Natl. Acad. Sci. U. S. A. 103, 744–749. 10.1073/pnas.0506643103 16407112 PMC1334643

[ref22] Ceravolo I. P. Chaves A. C. Bonjardim C. A. Sibley D. Romanha A. J. Gazzinelli R. T. (1999). Replication of *Toxoplasma gondii*, but not *Trypanosoma cruzi*, is regulated in human fibroblasts activated with gamma interferon: requirement of a functional JAK/STAT pathway. Infect. Immun. 67, 2233–2240. PMID: 10225879 10.1128/iai.67.5.2233-2240.1999 PMC115962

[ref23] Chang S. Shan X. Li X. Fan W. Zhang S. Q. Zhang J.. (2015). *Toxoplasma gondii* rhoptry protein ROP16 mediates partially SH-SY5Y cells apoptosis and cell cycle arrest by directing Ser15/37 phosphorylation of p53. Int. J. Biol. Sci. 11, 1215–1225. 10.7150/ijbs.10516, PMID: 26327815 PMC4551757

[ref24] Chen Z. W. Gao J. M. Huo X. X. Wang L. Yu L. Halm-Lai F.. (2011). Genotyping of *Toxoplasma gondii* isolates from cats in different geographic regions of China. Vet. Parasitol. 183, 166–170. 10.1016/j.vetpar.2011.06.013, PMID: 21757292

[ref25] Cirelli K. M. Gorfu G. Hassan M. A. Printz M. Crown D. Leppla S. H.. (2014). Inflammasome sensor NLRP1 controls rat macrophage susceptibility to *Toxoplasma gondii*. PLoS Pathog. 10:e1003927. 10.1371/journal.ppat.1003927, PMID: 24626226 PMC3953412

[ref26] Clough B. Frickel E. M. (2017). The *Toxoplasma* parasitophorous vacuole: an evolving host-parasite Frontier. Trends Parasitol. 33, 473–488. 10.1016/j.pt.2017.02.007, PMID: 28330745

[ref27] Curt-Varesano A. Braun L. Ranquet C. Hakimi M. A. Bougdour A. (2016). The aspartyl protease TgASP5 mediates the export of the *Toxoplasma* GRA16 and GRA24 effectors into host cells. Cell. Microbiol. 18, 151–167. 10.1111/cmi.12498, PMID: 26270241

[ref28] Darde M. L. Bouteille B. Pestre-Alexandre M. (1992). Isoenzyme analysis of 35 *Toxoplasma gondii* isolates and the biological and epidemiological implications. J. Parasitol. 78, 786–794. 10.2307/3283305, PMID: 1403418

[ref29] Deretic V. Saitoh T. Akira S. (2013). Autophagy in infection, inflammation and immunity. Nat. Rev. Immunol. 13, 722–737. 10.1038/nri3532, PMID: 24064518 PMC5340150

[ref30] Deter R. L. Baudhuin P. De Duve C. (1967). Participation of lysosomes in cellular autophagy induced in rat liver by glucagon. J. Cell Biol. 35, C11–C16. 10.1083/jcb.35.2.C11, PMID: 6055998 PMC2107130

[ref31] Di Cristina M. Dou Z. Lunghi M. Kannan G. Huynh M. H. McGovern O. L.. (2017). Toxoplasma depends on lysosomal consumption of autophagosomes for persistent infection. Nat. Microbiol. 2:17096. 10.1038/nmicrobiol.2017.96, PMID: 28628099 PMC5527684

[ref32] Du J. An R. Chen L. Shen Y. Chen Y. Cheng L.. (2014). *Toxoplasma gondii* virulence factor ROP18 inhibits the host NF-kappaB pathway by promoting p65 degradation. J. Biol. Chem. 289, 12578–12592. 10.1074/jbc.M113.544718, PMID: 24648522 PMC4007449

[ref33] Dubey J. P. (2010). Toxoplasmosis of animals and humans. 2nd edn (Boca Raton, Florida: CRC Press).

[ref34] Dubey J. P. Ferreira L. R. Alsaad M. Verma S. K. Alves D. A. Holland G. N.. (2016). Experimental toxoplasmosis in rats induced orally with eleven strains of *Toxoplasma gondii* of seven genotypes: tissue tropism, tissue cyst size, neural lesions, tissue cyst rupture without reactivation, and ocular lesions. PLoS One 11:e0156255. 10.1371/journal.pone.0156255, PMID: 27228262 PMC4882154

[ref35] Etheridge R. D. Alaganan A. Tang K. Lou H. J. Turk B. E. Sibley L. D. (2014). The *Toxoplasma* pseudokinase ROP5 forms complexes with ROP18 and ROP17 kinases that synergize to control acute virulence in mice. Cell Host Microbe 15, 537–550. 10.1016/j.chom.2014.04.002, PMID: 24832449 PMC4086214

[ref36] Frenal K. Polonais V. Marq J. B. Stratmann R. Limenitakis J. Soldati-Favre D. (2010). Functional dissection of the apicomplexan glideosome molecular architecture. Cell Host Microbe 8, 343–357. 10.1016/j.chom.2010.09.002, PMID: 20951968

[ref37] Gay G. Braun L. Brenier-Pinchart M. P. Vollaire J. Josserand V. Bertini R. L.. (2016). *Toxoplasma gondii* TgIST co-opts host chromatin repressors dampening STAT1-dependent gene regulation and IFN-gamma-mediated host defenses. J. Exp. Med. 213, 1779–1798. 10.1084/jem.20160340, PMID: 27503074 PMC4995087

[ref38] Gold D. A. Kaplan A. D. Lis A. Bett G. C. Rosowski E. E. Cirelli K. M.. (2015). The *Toxoplasma* dense granule proteins GRA17 and GRA23 mediate the movement of small molecules between the host and the parasitophorous vacuole. Cell Host Microbe 17, 642–652. 10.1016/j.chom.2015.04.003, PMID: 25974303 PMC4435723

[ref39] Gras S. Jackson A. Woods S. Pall G. Whitelaw J. Leung J. M.. (2017). Parasites lacking the micronemal protein MIC2 are deficient in surface attachment and host cell egress, but remain virulent in vivo. Wellcome Open Res. 2:32. 10.12688/wellcomeopenres.11594.1, PMID: 28630943 PMC5473411

[ref40] Graumann K. Schaumburg F. Reubold T. F. Hippe D. Eschenburg S. Luder C. G. (2015). Toxoplasma gondii inhibits cytochrome c-induced caspase activation in its host cell by interference with holo-apoptosome assembly. Microb Cell 2, 150–162. 10.15698/mic2015.05.201, PMID: 28357287 PMC5349237

[ref41] Guerin A. El Hajj H. Penarete-Vargas D. Besteiro S. Lebrun M. (2017). RON4L1 is a new member of the moving junction complex in *Toxoplasma gondii*. Sci. Rep. 7:17907. 10.1038/s41598-017-18010-9, PMID: 29263399 PMC5738351

[ref42] Hakimi M. A. Olias P. Sibley L. D. (2017). Toxoplasma effectors targeting host signaling and transcription. Clin. Microbiol. Rev. 30, 615–645. 10.1128/cmr.00005-17, PMID: 28404792 PMC5475222

[ref43] Haldar A. K. Piro A. S. Pilla D. M. Yamamoto M. Coers J. (2014). The E2-like conjugation enzyme Atg3 promotes binding of IRG and Gbp proteins to Chlamydia- and Toxoplasma-containing vacuoles and host resistance. PLoS One 9:e86684. 10.1371/journal.pone.0086684, PMID: 24466199 PMC3895038

[ref44] Hermanns T. Muller U. B. Konen-Waisman S. Howard J. C. Steinfeldt T. (2016). The *Toxoplasma gondii* rhoptry protein ROP18 is an Irga6-specific kinase and regulated by the dense granule protein GRA7. Cell. Microbiol. 18, 244–259. 10.1111/cmi.12499, PMID: 26247512 PMC5061101

[ref45] Herm-Gotz A. Weiss S. Stratmann R. Fujita-Becker S. Ruff C. Meyhofer E.. (2002). *Toxoplasma gondii* myosin A and its light chain: a fast, single-headed, plus-end-directed motor. EMBO J. 21, 2149–2158. 10.1093/emboj/21.9.2149, PMID: 11980712 PMC125985

[ref46] Hippe D. Lytovchenko O. Schmitz I. Luder C. G. (2008). Fas/CD95-mediated apoptosis of type II cells is blocked by Toxoplasma gondii primarily via interference with the mitochondrial amplification loop. Infect. Immun. 76, 2905–2912. 10.1128/IAI.01546-07, PMID: 18411295 PMC2446730

[ref47] Hong D. P. Radke J. B. White M. W. (2017). Opposing transcriptional mechanisms regulate *Toxoplasma* development. mSphere 2:e00347. 10.1128/mSphere.00347-16, PMID: 28251183 PMC5322347

[ref48] Howard J. C. Hunn J. P. Steinfeldt T. (2011). The IRG protein-based resistance mechanism in mice and its relation to virulence in Toxoplasma gondii. Curr. Opin. Microbiol. 14, 414–421. 10.1016/j.mib.2011.07.002, PMID: 21783405

[ref49] Howe D. K. Honore S. Derouin F. Sibley L. D. (1997). Determination of genotypes of *Toxoplasma gondii* strains isolated from patients with toxoplasmosis. J. Clin. Microbiol. 35, 1411–1414. PMID: 9163454 10.1128/jcm.35.6.1411-1414.1997 PMC229759

[ref50] Howe D. K. Sibley L. D. (1995). Toxoplasma gondii comprises three clonal lineages: correlation of parasite genotype with human disease. J. Infect. Dis. 172, 1561–1566. 10.1093/infdis/172.6.1561, PMID: 7594717

[ref51] Huang S. Holmes M. J. Radke J. B. Hong D. P. Liu T. K. White M. W.. (2017). *Toxoplasma gondii* AP2IX-4 regulates gene expression during bradyzoite development. mSphere 2:e00054. 10.1128/mSphere.00054-17, PMID: 28317026 PMC5352832

[ref52] Hwang I. Y. Quan J. H. Ahn M. H. Ahmed H. A. Cha G. H. Shin D. W.. (2010). Toxoplasma gondii infection inhibits the mitochondrial apoptosis through induction of Bcl-2 and HSP70. Parasitol. Res. 107, 1313–1321. 10.1007/s00436-010-1999-3, PMID: 20680337

[ref53] Jacot D. Tosetti N. Pires I. Stock J. Graindorge A. Hung Y. F.. (2016). An apicomplexan actin-binding protein serves as a connector and lipid sensor to coordinate motility and invasion. Cell Host Microbe 20, 731–743. 10.1016/j.chom.2016.10.020, PMID: 27978434

[ref54] Jewett T. J. Sibley L. D. (2003). Aldolase forms a bridge between cell surface adhesins and the actin cytoskeleton in apicomplexan parasites. Mol. Cell 11, 885–894. 10.1016/S1097-2765(03)00113-8, PMID: 12718875

[ref55] Johnston A. C. Piro A. Clough B. Siew M. Virreira Winter S. Coers J.. (2016). Human GBP1 does not localize to pathogen vacuoles but restricts *Toxoplasma gondii*. Cell. Microbiol. 18, 1056–1064. 10.1111/cmi.12579, PMID: 26874079 PMC4961618

[ref56] Kim J. Kundu M. Viollet B. Guan K. L. (2011). AMPK and mTOR regulate autophagy through direct phosphorylation of Ulk1. Nat. Cell Biol. 13, 132–141. 10.1038/ncb2152, PMID: 21258367 PMC3987946

[ref57] Kim J. H. Lee J. Bae S. J. Kim Y. Park B. J. Choi J. W. (2017). NADPH oxidase 4 is required for the generation of macrophage migration inhibitory factor and host defense against *Toxoplasma gondii* infection. Sci. Rep. 7:6361. 10.1038/s41598-017-06610-4 28743960 PMC5526938

[ref58] Koblansky A. A. Jankovic D. Oh H. Hieny S. Sungnak W. Mathur R.. (2013). Recognition of profilin by toll-like receptor 12 is critical for host resistance to *Toxoplasma gondii*. Immunity 38, 119–130. 10.1016/j.immuni.2012.09.016, PMID: 23246311 PMC3601573

[ref59] Kwok L. Y. Schlüter D. Clayton C. Soldati D. (2003). The antioxidant systems in *Toxoplasma gondii* and the role of cytosolic catalase in defence against oxidative injury. Mol. Microbiol. 51, 47–61. 10.1046/j.1365-2958.2003.03823.x 14651610

[ref60] Lai S. C. Devenish R. J. (2012). LC3-associated phagocytosis (LAP): connections with host autophagy. Cell 1, 396–408. 10.3390/cells1030396, PMID: 24710482 PMC3901117

[ref61] Lamarque M. Besteiro S. Papoin J. Roques M. Vulliez-Le Normand B. Morlon-Guyot J.. (2011). The RON2-AMA1 interaction is a critical step in moving junction-dependent invasion by apicomplexan parasites. PLoS Pathog. 7:e1001276. 10.1371/journal.ppat.1001276, PMID: 21347343 PMC3037350

[ref62] Lamarque M. H. Roques M. Kong-Hap M. Tonkin M. L. Rugarabamu G. Marq J. B.. (2014). Plasticity and redundancy among AMA-RON pairs ensure host cell entry of *Toxoplasma* parasites. Nat. Commun. 5:4098. 10.1038/ncomms5098, PMID: 24934579

[ref63] Lebrun M. Michelin A. El Hajj H. Poncet J. Bradley P. J. Vial H.. (2005). The rhoptry neck protein RON4 re-localizes at the moving junction during *Toxoplasma gondii* invasion. Cell. Microbiol. 7, 1823–1833. 10.1111/j.1462-5822.2005.00646.x, PMID: 16309467

[ref64] Li J. X. He J. J. Elsheikha H. M. Chen D. Zhai B. T. Zhu X. Q.. (2019). *Toxoplasma gondii* ROP17 inhibits the innate immune response of HEK293T cells to promote its survival. Parasitol. Res. 118, 783–792. 10.1007/s00436-019-06215-y, PMID: 30675671

[ref65] Li M. Mo X. W. Wang L. Chen H. Luo Q. L. Wen H. Q. (2014). Phylogeny and virulence divergency analyses of *Toxoplasma gondii* isolates from China. Parasit. Vectors 7:133. 10.1186/1756-3305-7-133 24678633 PMC3986613

[ref66] Liu E. Lopez Corcino Y. Portillo J. A. Miao Y. Subauste C. S. (2016). Identification of signaling pathways by which CD40 stimulates autophagy and antimicrobial activity against *Toxoplasma gondii* in macrophages. Infect. Immun. 84, 2616–2626. 10.1128/IAI.00101-16, PMID: 27354443 PMC4995900

[ref67] Luder C. G. Gross U. Lopes M. F. (2001). Intracellular protozoan parasites and apoptosis: diverse strategies to modulate parasite-host interactions. Trends Parasitol. 17, 480–486. 10.1016/S1471-4922(01)02016-5, PMID: 11587962

[ref68] Ma J. S. Sasai M. Ohshima J. Lee Y. Bando H. Takeda K.. (2014). Selective and strain-specific NFAT4 activation by the *Toxoplasma gondii* polymorphic dense granule protein GRA6. J. Exp. Med. 211, 2013–2032. 10.1084/jem.20131272, PMID: 25225460 PMC4172224

[ref69] Matta S. K. Patten K. Wang Q. Kim B. H. MacMicking J. D. Sibley L. D. (2018). NADPH oxidase and guanylate binding protein 5 restrict survival of avirulent type III strains of *Toxoplasma gondii* in naive macrophages. MBio 9:e01393. 10.1128/mBio.01393-18, PMID: 30154263 PMC6113620

[ref70] Meissner M. Reiss M. Viebig N. Carruthers V. B. Toursel C. Tomavo S. (2002a). A family of transmembrane microneme proteins of Toxoplasma gondii contain EGF-like domains and function as escorters. J. Cell Sci. 115, 563–574. 10.1116/1.584155 11861763

[ref71] Meissner M. Schluter D. Soldati D. (2002b). Role of *Toxoplasma gondii* myosin A in powering parasite gliding and host cell invasion. Science 298, 837–840. 10.1126/science.1074553 12399593

[ref72] Mizushima N. Yoshimori T. Ohsumi Y. (2011). The role of Atg proteins in autophagosome formation. Annu. Rev. Cell Dev. Biol. 27, 107–132. 10.1146/annurev-cellbio-092910-154005, PMID: 21801009

[ref73] Mordue D. Sibley D. (1997). Intracellular fate of vacuoles containing *Toxoplasma gondii* is determined at the time of formation and depends on the mechanism of entry. J. Immunol. 9, 4452–4459. 9379044

[ref74] Morgado P. Sudarshana D. M. Gov L. Harker K. S. Lam T. Casali P.. (2014). Type II *Toxoplasma gondii* induction of CD40 on infected macrophages enhances interleukin-12 responses. Infect. Immun. 82, 4047–4055. 10.1128/IAI.01615-14, PMID: 25024369 PMC4187859

[ref75] Moroda M. Takamoto M. Iwakura Y. Nakayama J. Aosai F. (2017). Interleukin-17A-deficient mice are highly susceptible to *Toxoplasma gondii* infection due to excessively induced *T. gondii* HSP70 and interferon gamma production. Infect. Immun. 85:e00399-17. 10.1128/IAI.00399-17, PMID: 28893913 PMC5695131

[ref76] Muniz-Feliciano L. Van Grol J. Portillo J. A. Liew L. Liu B. Carlin C. R.. (2013). *Toxoplasma gondii*-induced activation of EGFR prevents autophagy protein-mediated killing of the parasite. PLoS Pathog. 9:e1003809. 10.1371/journal.ppat.1003809, PMID: 24367261 PMC3868508

[ref77] Nagineni C. N. Pardhasaradhi K. Martins M. C. Detrick B. Hooks J. J. (1996). Mechanisms of interferon-induced inhibition of Toxoplasma gondii replication in human retinal pigment epithelial cells. Infect. Immun. 64, 4188–4196. PMID: 8926087 10.1128/iai.64.10.4188-4196.1996 PMC174355

[ref78] Ngo H. M. Zhou Y. Lorenzi H. Wang K. Kim T. K. Zhou Y. (2017). Toxoplasma modulates signature pathways of human epilepsy, neurodegeneration & cancer. Sci. Rep. 7:11496. 10.1038/s41598-017-10675-6 28904337 PMC5597608

[ref79] Niedelman W. Gold D. A. Rosowski E. E. Sprokholt J. K. Lim D. Farid Arenas A.. (2012). The rhoptry proteins ROP18 and ROP5 mediate *Toxoplasma gondii* evasion of the murine, but not the human, interferon-gamma response. PLoS Pathog. 8:e1002784. 10.1371/journal.ppat.1002784, PMID: 22761577 PMC3386190

[ref80] Nishikawa Y. Kawase O. Vielemeyer O. Suzuki H. Joiner K. A. Xuan X. (2007). Toxoplasma gondii infection induces apoptosis in noninfected macrophages: role of nitric oxide and other soluble factors. Parasite Immunol. 29, 375–385. 10.1111/j.1365-3024.2007.00956.x 17576367

[ref81] Ohshima J. Lee Y. Sasai M. Saitoh T. Su Ma J. Kamiyama N.. (2014). Role of mouse and human autophagy proteins in IFN-gamma-induced cell-autonomous responses against *Toxoplasma gondii*. J. Immunol. 192, 3328–3335. 10.4049/jimmunol.1302822, PMID: 24563254

[ref82] Ohsumi Y. (2014). Historical landmarks of autophagy research. Cell Res. 24, 9–23. 10.1038/cr.2013.169, PMID: 24366340 PMC3879711

[ref83] Olias P. Etheridge R. D. Zhang Y. Holtzman M. J. Sibley L. D. (2016). Toxoplasma effector recruits the Mi-2/NuRD complex to repress STAT1 transcription and block IFN-gamma-dependent gene expression. Cell Host Microbe 20, 72–82. 10.1016/j.chom.2016.06.006, PMID: 27414498 PMC4947229

[ref84] Padrao Jda C. Cabral G. R. da Silva Mde F. Seabra S. H. DaMatta R. A. (2014). *Toxoplasma gondii* infection of activated J774-A1 macrophages causes inducible nitric oxide synthase degradation by the proteasome pathway. Parasitol. Int. 63, 659–663. 10.1016/j.parint.2014.05.003, PMID: 24845536

[ref85] Parker M. L. Penarete-Vargas D. M. Hamilton P. T. Guerin A. Dubey J. P. Perlman S. J.. (2016). Dissecting the interface between apicomplexan parasite and host cell: insights from a divergent AMA-RON2 pair. Proc. Natl. Acad. Sci. U. S. A. 113, 398–403. 10.1073/pnas.1515898113, PMID: 26712012 PMC4720339

[ref86] Pavlou G. Biesaga M. Touquet B. Lagal V. Balland M. Dufour A.. (2018). Toxoplasma parasite twisting motion mechanically induces host cell membrane fission to complete invasion within a protective vacuole. Cell Host Microbe 24, 81–96.e85. 10.1016/j.chom.2018.06.003, PMID: 30008293

[ref87] Peixoto L. Chen F. Harb O. S. Davis P. H. Beiting D. P. Brownback C. S.. (2010). Integrative genomic approaches highlight a family of parasite-specific kinases that regulate host responses. Cell Host Microbe 8, 208–218. 10.1016/j.chom.2010.07.004, PMID: 20709297 PMC2963626

[ref88] Pfefferkorn E. R. Eckel M. Rebhun S. (1986). Interferon-gamma suppresses the growth of Toxoplasma gondii in human fibroblasts through starvation for tryptophan. Mol. Biochem. Parasitol. 20, 215–224. 10.1016/0166-6851(86)90101-5, PMID: 3093859

[ref89] Pino P. Foth B. J. Kwok L. Y. Sheiner L. Schepers R. Soldati T.. (2007). Dual targeting of antioxidant and metabolic enzymes to the mitochondrion and the apicoplast of Toxoplasma gondii. PLoS Pathog. 3:e115. 10.1371/journal.ppat.0030115, PMID: 17784785 PMC1959373

[ref90] Portillo J. C. Muniz-Feliciano L. Lopez Corcino Y. Lee S. J. Van Grol J. Parsons S. J. (2017). *Toxoplasma gondii* induces FAK-Src-STAT3 signaling during infection of host cells that prevents parasite targeting byautophagy. PLoS Pathog. 13:e1006671. 10.1371/journal.ppat.1006671 29036202 PMC5658194

[ref91] Portillo J. A. Okenka G. Reed E. Subauste A. Van Grol J. Gentil K.. (2010). The CD40-autophagy pathway is needed for host protection despite IFN-gamma-dependent immunity and CD40 induces autophagy via control of P21 levels. PLoS One 5:e14472. 10.1371/journal.pone.0014472, PMID: 21217818 PMC3013095

[ref92] Poukchanski A. Fritz H. M. Tonkin M. L. Treeck M. Boulanger M. J. Boothroyd J. C. (2013). *Toxoplasma gondii* sporozoites invade host cells using two novel paralogues of RON2 and AMA1. PLoS One 8:e70637. 10.1371/journal.pone.0070637, PMID: 23940612 PMC3734201

[ref93] Quan J. H. Cha G. H. Zhou W. Chu J. Q. Nishikawa Y. Lee Y. H. (2013). Involvement of PI 3 kinase/Akt-dependent Bad phosphorylation in *Toxoplasma gondii*-mediated inhibition of host cell apoptosis. Exp. Parasitol. 133, 462–471. 10.1016/j.exppara.2013.01.005, PMID: 23333591

[ref94] Rabenau K. E. Sohrabi A. Tripathy A. Reitter C. Ajioka J. W. Tomley F. M.. (2001). TgM2AP participates in *Toxoplasma gondii* invasion of host cells and is tightly associated with the adhesive protein TgMIC2. Mol. Microbiol. 41, 537–547. 10.1046/j.1365-2958.2001.02513.x, PMID: 11532123

[ref95] Roach J. C. Glusman G. Rowen L. Kaur A. Purcell M. K. Smith K. D. (2005). The evolution of vertebrate toll-like receptors. Proc. Natl. Acad. Sci. U. S. A. 102, 9577–9582. 10.1073/pnas.0502272102 15976025 PMC1172252

[ref96] Rosowski E. E. Saeij J. P. (2012). *Toxoplasma gondii* clonal strains all inhibit STAT1 transcriptional activity but polymorphic effectors differentially modulate IFNgamma induced gene expression and STAT1 phosphorylation. PLoS One 7:e51448. 10.1371/journal.pone.0051448, PMID: 23240025 PMC3519884

[ref97] Safronova A. Araujo A. Camanzo E. T. Moon T. J. Elliott M. R. Beiting D. P.. (2019). Alarmin S100A11 initiates a chemokine response to the human pathogen *Toxoplasma gondii*. Nat. Immunol. 20, 64–72. 10.1038/s41590-018-0250-8, PMID: 30455460 PMC6291348

[ref98] Sasai M. Sakaguchi N. Ma J. S. Nakamura S. Kawabata T. Bando H.. (2017). Essential role for GABARAP autophagy proteins in interferon-inducible GTPase-mediated host defense. Nat. Immunol. 18, 899–910. 10.1038/ni.3767, PMID: 28604719

[ref99] Selleck E. M. Orchard R. C. Lassen K. G. Beatty W. L. Xavier R. J. Levine B.. (2015). A noncanonical autophagy pathway restricts *Toxoplasma gondii* growth in a strain-specific manner in IFN-gamma-activated human cells. MBio 6, e01157–e01115. 10.1128/mBio.01157-15, PMID: 26350966 PMC4600106

[ref100] Sergent V. Cautain B. Khalife J. Deslee D. Bastien P. Dao A.. (2005). Innate refractoriness of the Lewis rat to toxoplasmosis is a dominant trait that is intrinsic to bone marrow-derived cells. Infect. Immun. 73, 6990–6997. 10.1128/iai.73.10.6990-6997.2005, PMID: 16177379 PMC1230985

[ref101] Sibley L. D. Boothroyd J. C. (1992). Virulent strains of *Toxoplasma gondii* comprise a single clonal lineage. Nature 359, 82–85. 10.1038/359082a0, PMID: 1355855

[ref102] Sibley L. D. Khan A. Ajioka J. W. Rosenthal B. M. (2009). Genetic diversity of *Toxoplasma gondii* in animals and humans. Philos. Trans. R. Soc. Lond. Ser. B Biol. Sci. 364, 2749–2761. 10.1098/rstb.2009.0087 19687043 PMC2865090

[ref103] Soldati D. Dubremetz J. F. Lebrun M. (2001). Microneme proteins: structural and functional requirements to promote adhesion and invasion by the apicomplexan parasite *Toxoplasma gondii*. Int. J. Parasitol. 31, 1293–1302. 10.1016/S0020-7519(01)00257-0, PMID: 11566297

[ref104] Song K. J. Ahn H. J. Nam H. W. (2012). Anti-apoptotic effects of SERPIN B3 and B4 via STAT6 activation in macrophages after infection with Toxoplasma gondii. Korean J. Parasitol. 50, 1–6. 10.3347/kjp.2012.50.1.1, PMID: 22451727 PMC3309045

[ref105] Tahara M. Andrabi S. B. Matsubara R. Aonuma H. Nagamune K. (2016). A host cell membrane microdomain is a critical factor for organelle discharge by *Toxoplasma gondii*. Parasitol. Int. 65, 378–388. 10.1016/j.parint.2016.05.012, PMID: 27217289

[ref106] Tomita T. Bzik D. J. Ma Y. F. Fox B. A. Markillie L. M. Taylor R. C.. (2013). The Toxoplasma gondii cyst wall protein CST1 is critical for cyst wall integrity and promotes bradyzoite persistence. PLoS Pathog. 9:e1003823. 10.1371/journal.ppat.1003823, PMID: 24385904 PMC3873430

[ref107] Tomita T. Sugi T. Yakubu R. Tu V. Ma Y. Weiss L. M. (2017). Making home sweet and sturdy: *Toxoplasma gondii* ppGalNAc-Ts glycosylate in hierarchical order and confer cyst wall rigidity. MBio 8:e02048-16. 10.1128/mBio.02048-16, PMID: 28074022 PMC5225312

[ref108] Tonkin M. L. Roques M. Lamarque M. H. Pugniere M. Douguet D. Crawford J.. (2011). Host cell invasion by apicomplexan parasites: insights from the co-structure of AMA1 with a RON2 peptide. Science 333, 463–467. 10.1126/science.1204988, PMID: 21778402

[ref109] Tyler J. S. Boothroyd J. C. (2011). The C-terminus of *Toxoplasma* RON2 provides the crucial link between AMA1 and the host-associated invasion complex. PLoS Pathog. 7:e1001282. 10.1371/journal.ppat.1001282, PMID: 21347354 PMC3037364

[ref110] Van Grol J. Muniz-Feliciano L. Portillo J. A. Bonilha V. L. Subauste C. S. (2013). CD40 induces anti-Toxoplasma gondii activity in nonhematopoietic cells dependent on autophagy proteins. Infect. Immun. 81, 2002–2011. 10.1128/iai.01145-12, PMID: 23509150 PMC3676008

[ref111] Wang L. Chen H. Liu D. Huo X. Gao J. Song X.. (2013). Genotypes and mouse virulence of *Toxoplasma gondii* isolates from animals and humans in China. PLoS One 8:e53483. 10.1371/journal.pone.0053483, PMID: 23308233 PMC3538538

[ref112] Wang Y. Cirelli K. M. Barros P. D. C. Sangare L. O. Butty V. Hassan M. A.. (2019). Three *Toxoplasma gondii* dense granule proteins are required for induction of Lewis rat macrophage pyroptosis. MBio 10:e02388-18. 10.1128/mBio.02388-18, PMID: 30622189 PMC6325250

[ref113] Wang Y. Fang R. Yuan Y. Pan M. Hu M. Zhou Y. (2016). Identification of host proteins, Spata3 and Dkk2, interacting with *Toxoplasma gondii* micronemal protein MIC3. Parasitol. Res. 115, 2825–2835. 10.1007/s00436-016-5033-2 27053129

[ref114] Wang S. Hassan I. A. Liu X. Xu L. Yan R. Song X.. (2015). Immunological changes induced by *Toxoplasma gondii* glutathione-S-transferase (TgGST) delivered as a DNA vaccine. Res. Vet. Sci. 99, 157–164. 10.1016/j.rvsc.2014.12.006, PMID: 25648285

[ref115] Wang Y. Yin H. (2015). Research advances in microneme protein 3 of *Toxoplasma gondii*. Parasit. Vectors 8:384. 10.1186/s13071-015-1001-4, PMID: 26194005 PMC4509771

[ref116] Wang S. Zhang Z. Wang Y. Gadahi J. A. Xie Q. Xu L.. (2017). *Toxoplasma gondii* excretory/secretory antigens (TgESAs) suppress pro-inflammatory cytokine secretion by inhibiting TLR-induced NF-kappaB activation in LPS-stimulated murine macrophages. Oncotarget 8, 88351–88359. 10.18632/oncotarget.19362, PMID: 29179440 PMC5687610

[ref117] Wu L. Wang X. Li Y. Liu Y. Su D. Fu T.. (2016). *Toxoplasma gondii* ROP18: potential to manipulate host cell mitochondrial apoptosis. Parasitol. Res. 115, 2415–2422. 10.1007/s00436-016-4993-6, PMID: 27021182

[ref118] Xia J. Kong L. Zhou L. J. Wu S. Z. Yao L. J. He C.. (2018). Genome-wide bimolecular fluorescence complementation-based proteomic analysis of *Toxoplasma gondii* ROP18's human interactome shows its key role in regulation of cell immunity and apoptosis. Front. Immunol. 9:61. 10.3389/fimmu.2018.00061, PMID: 29459857 PMC5807661

[ref119] Xue J. Jiang W. Chen Y. Gong F. Wang M. Zeng P.. (2017). Thioredoxin reductase from *Toxoplasma gondii*: an essential virulence effector with antioxidant function. FASEB J. 31, 4447–4457. 10.1096/fj.201700008R, PMID: 28687608

[ref120] Yamamoto M. Ma J. S. Mueller C. Kamiyama N. Saiga H. Kubo E.. (2011). ATF6beta is a host cellular target of the *Toxoplasma gondii* virulence factor ROP18. J. Exp. Med. 208, 1533–1546. 10.1084/jem.20101660, PMID: 21670204 PMC3135360

[ref121] Yamamoto M. Standley D. M. Takashima S. Saiga H. Okuyama M. Kayama H.. (2009). A single polymorphic amino acid on *Toxoplasma gondii* kinase ROP16 determines the direct and strain-specific activation of Stat3. J. Exp. Med. 206, 2747–2760. 10.1084/jem.20091703, PMID: 19901082 PMC2806617

[ref122] Yamamoto M. Takeda K. (2012). Inhibition of ATF6beta-dependent host adaptive immune response by a *Toxoplasma* virulence factor ROP18. Virulence 3, 77–80. 10.4161/viru.3.1.18340 22286708

[ref123] Yang Z. Hou Y. Hao T. Rho H. S. Wan J. Luan Y. (2017b). A human proteome array approach to identifying key host proteins targeted by *Toxoplasma* kinase ROP18. Mol. Cell. Proteomics 16, 469–484. 10.1074/mcp.M116.063602 28087594 PMC5341007

[ref124] Yang W. B. Zhou D. H. Zou Y. Chen K. Liu Q. Wang J. L. (2017a). Vaccination with a DNA vaccine encoding *Toxoplasma gondii* ROP54 induces protective immunity against toxoplasmosis in mice. Acta Trop. 176, 427–432. 10.1016/j.actatropica.2017.09.007 28935555

[ref125] Yarovinsky F. Zhang D. Andersen J. F. Bannenberg G. L. Serhan C. N. Hayden M. S.. (2005). TLR11 activation of dendritic cells by a protozoan profilin-like protein. Science 308, 1626–1629. 10.1126/science.1109893, PMID: 15860593

[ref126] Zhao Z. Fux B. Goodwin M. Dunay I. R. Strong D. Miller B. C.. (2008). Autophagosome-independent essential function for the autophagy protein Atg5 in cellular immunity to intracellular pathogens. Cell Host Microbe 4, 458–469. 10.1016/j.chom.2008.10.003, PMID: 18996346 PMC2682425

[ref127] Zhou P. Nie H. Zhang L. X. Wang H. Y. Yin C. C. Su C.. (2010). Genetic characterization of *Toxoplasma gondii* isolates from pigs in China. J. Parasitol. 96, 1027–1029. 10.1645/GE-2465.1, PMID: 20481661

[ref128] Zhou P. Sun X. T. Yin C. C. Yang J. F. Yuan Z. G. Yan H. K.. (2011). Genetic characterization of *Toxoplasma gondii* isolates from pigs in southwestern China. J. Parasitol. 97, 1193–1195. 10.1645/ge-2851.1, PMID: 21721904

